# Motor Unit Number Estimation for Evaluating Disease Progression and Comparison With Functional Rating Scale Scores in Patients With Amyotrophic Lateral Sclerosis

**DOI:** 10.7759/cureus.85348

**Published:** 2025-06-04

**Authors:** Divya Rani, Shishir K Chandan, Ekta Devi

**Affiliations:** 1 Neurology, Vardhman Mahavir Medical College and Safdarjung Hospital, New Delhi, IND; 2 Neurology, Vardhman Mahavir Medical College and Safdarjung Hospital, New delhi, IND

**Keywords:** amyotrophic lateral sclerosis (als), amyotrophic lateral sclerosis functional rating scale–revised (alsfrs-r), motor unit action potential, motor unit number estimation (mune), multi-point stimulation, prognostic marker in als, severity of als, single motor unit potential

## Abstract

Introduction

Amyotrophic lateral sclerosis (ALS) is a progressive motor neuron disease characterized by degeneration of motor neurons in the brain, brainstem, and spinal cord. While the ALS Functional Rating Scale-Revised (ALSFRS-R) is commonly used to assess functional decline, its limitations highlight the need for more objective biomarkers. Motor unit number estimation (MUNE) is an electrophysiological technique that may provide a more sensitive measure of disease progression. This study aims to evaluate the utility of MUNE as a biomarker in ALS and compare its performance with ALSFRS-R.

Methods

This was a prospective, single-center, observational study conducted over 18 months. A total of 31 patients with definite or probable ALS, diagnosed per the Revised El Escorial Criteria, were enrolled. MUNE and ALSFRS-R assessments were performed at baseline and after six months. MUNE was calculated using the multi-point incremental method in the upper extremity. Data were analyzed using paired t-tests and Pearson’s correlation coefficients. Subgroup analyses by age, sex, and symptom onset site were also conducted.

Results

Both MUNE and ALSFRS-R scores declined significantly over six months. The mean MUNE decreased from 16.36 ± 5.22 to 13.37 ± 4.96 (p < 0.0001), while the ALSFRS-R score declined from 42.06 ± 3.24 to 36.72 ± 4.89 (p < 0.0001). The mean rate of decline was significantly greater for MUNE (23.6 ± 15.31) than for ALSFRS-R (13.91 ± 8.18; p = 0.001). No significant associations were observed between MUNE and patient age, sex, or site of symptom onset. Correlation between MUNE and ALSFRS-R was weak at both time points.

Conclusions

MUNE demonstrated a significantly greater rate of decline than ALSFRS-R, suggesting higher sensitivity to motor neuron loss over time. These findings support using MUNE as a reliable and objective biomarker for monitoring disease progression in ALS. Incorporating MUNE into clinical practice and research may improve prognostication, enable earlier therapeutic intervention, and enhance patient stratification in clinical trials. Further large-scale studies are needed to validate its routine use.

## Introduction

Motor neuron diseases (MNDs) comprise a spectrum of neurodegenerative disorders characterized by progressive muscle weakness due to the degeneration of motor neurons in the brain, brainstem, and spinal cord. Amyotrophic lateral sclerosis (ALS) is the most common form of MND in adults [1]. ALS is a progressive neurodegenerative condition, with most patients dying within three to five years of diagnosis [2]. Once the diagnosis of ALS is established, prognostic assessment becomes critical to help patients make informed decisions regarding their treatment preferences and end-of-life planning.

The ALS Functional Rating Scale-Revised (ALSFRS-R) is the most widely used tool to assess functional ability in patients with ALS [3]. Although it is easy to administer and standardized, the ALSFRS-R is not unidimensional; it includes items that evaluate domains beyond motor function, limiting its reliability as a sole outcome measure [4].

Motor unit number estimation (MUNE) is an electrophysiological technique used to estimate the approximate number of functioning lower motor neurons innervating a specific muscle or muscle group. MUNE studies have demonstrated the progressive spread of ALS within and between regions [5]. Motor unit numbers often decline before clinical weakness becomes apparent, making MUNE a useful surrogate marker in clinical trials [6]. Unlike other techniques, MUNE quantitatively estimates motor unit counts and is not confounded by collateral reinnervation. Therefore, MUNE serves as a reliable, physiologic method to quantify motor unit loss and monitor disease progression [7,8]. This study aims to compare the utility of MUNE as a biomarker in ALS versus ALSFRS-R.

## Materials and methods

Study design

This was a single-center, prospective, hospital-based observational study conducted over 18 months (September 2022 to February 2024). Patients diagnosed with definite or probable ALS based on the Revised El Escorial Criteria [9] were included. ALSFRS-R scores and MUNE values were assessed at baseline and at six months. The rate of decline over six months was calculated and compared between the two metrics. The Institutional Review Board/Thesis Protocol Review Committee of Vardhman Mahavir Medical College and Safdarjung Hospital approved the study design (approval no. IEC/VMMC/SJH/Thesis/9/2022/CC-11).

MUNE method

The multi-point incremental method was used to calculate MUNE [10]. Either the median or ulnar nerve in one upper extremity was selected for study. Surface recording electrodes were placed on the abductor pollicis brevis (innervated by the median nerve) or the abductor digiti minimi (innervated by the ulnar nerve) using a standard belly-tendon montage.

For the median nerve, stimulation was applied at three locations: 2 cm proximal to the wrist crease, 4 cm proximal to the first site, and in the cubital fossa [10]. For the ulnar nerve, stimulation sites included 2 cm proximal to the wrist crease, 4 cm proximal to the first site, and 1 cm proximal to the ulnar groove at the elbow [10]. Filter settings were set to 10 Hz-10 kHz.

At each site, the optimal stimulation point was identified using a submaximal stimulus to elicit the greatest response. Marked sites were then stimulated using self-adhesive circular electrodes. A maximal response was obtained at the most distal site. Amplifier gain was then adjusted to 50 µV/division, and the stimulus control was set to the maximum level.

Using a standard three-site motor conduction protocol, subthreshold stimuli were incrementally increased at approximately 4 mA/s until a clearly defined all-or-nothing response (>25 µV) was recorded (trace 1). Further incremental responses were elicited and recorded as traces 2 and 3. The negative peak amplitude of each third response was recorded. This procedure was repeated at the second and third stimulation sites.

The sum of the third response amplitudes across the three sites was calculated and divided by nine to yield the average single motor unit potential (SMUP) amplitude. Maximum compound muscle action potential (CMAP) amplitude was then divided by SMUP to calculate the MUNE.

During the initial visit, the upper extremities were assessed clinically. If both arms exhibited weakness, the stronger arm was selected. If only one arm was weak, it was chosen for evaluation. Median nerve conduction (motor and sensory) studies were performed to exclude carpal tunnel syndrome. The ulnar nerve was assessed if a median neuropathy or a CMAP amplitude of <5 mV was identified. The contralateral limb was evaluated to determine if both nerves were affected or had CMAP amplitudes <5 mV. A nerve-muscle pair free of focal neuropathy and with a low-normal CMAP amplitude was prioritized. If all tested nerves exhibited reduced CMAP amplitudes, the one with the highest amplitude was used.

MUNE calculation example

**Figure 1:**
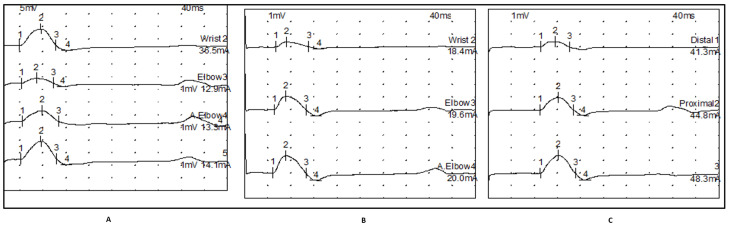
(A) CMAP and motor unit potential after three incremental stimulations at the wrist. (B) Motor unit potential after three incremental stimulations 4 cm above the wrist. (C) Motor unit potential after three incremental stimulations at the elbow. CMAP, compound muscle action potential

Table 1 and Figure 1 illustrate the calculation of MUNE in an ALS patient. Three incremental stimuli were delivered at the wrist, 4 cm proximal to the wrist, and at the elbow, yielding third response amplitudes of 1.4, 1.5, and 1.6 mV, respectively. The sum of these values (4.5 mV) was divided by nine to obtain the SMUP amplitude (0.5 mV). The CMAP amplitude (6.5 mV) was then divided by the SMUP amplitude to calculate the MUNE: MUNE = 6.5 / 0.5 = 13.

**Table 1:** CMAP with MUNE values of three responses each at three different sites in tabulated form APB, abductor pollicis brevis; CMAP, compound muscle action potential; MUNE, motor unit number estimation

Nerve/Sites		Recorded site	Latency, ms	Amplitude, mV
			ms	mV
CMAP		APB	3.96	6.5
L median - APB (at the wrist)	1st Response	APB	4.27	0.4
	2nd response	APB	4.38	0.8
	3rd response	APB	4.17	1.4
L median - APB (4 cm above the wrist)	1st response	APB	5.42	0.5
	2nd response	APB	5.10	0.9
	3rd response	APB	4.84	1.5
L median - APB (at the elbow)	1st response	APB	9.11	0.6
	2nd response	APB	9.01	1.0
	3rd response	APB	9.06	1.6

Statistical analysis

Categorical variables were presented as frequencies and percentages. Continuous variables were reported as means ± standard deviation (SD) or as medians with interquartile ranges (25th and 75th percentiles), as appropriate. Data normality was assessed using the Shapiro-Wilk test.

Comparisons of continuous variables were performed using paired t-tests. Correlation analyses between ALSFRS-R scores and MUNE values and their respective rates of decline were conducted using Pearson’s correlation coefficient. Data were entered into Microsoft Excel and analyzed using IBM SPSS Statistics for Windows, Version 25.0. (Armonk, NY: IBM Corp.). A two-tailed p-value of <0.05 was considered statistically significant.

## Results

The mean age of presentation in our cohort was 49 years. The male-to-female ratio was 1.5:1. Of the 35 patients enrolled, 30 (85.71%) patients presented with spinal-onset symptoms, while five (14.29%) patients had bulbar-onset symptoms. The mean duration of symptoms was 14 months in the spinal group and six months in the bulbar group.

Eight (22.86%) patients could not perform pulmonary function testing due to poor effort. Most of these patients (60%) had bulbar-onset symptoms, suggesting a potential association between bulbar involvement and poor respiratory effort during testing.

At baseline, the mean CMAP amplitude was 7.57 mV. After six months, the mean CMAP had decreased to 5.64 mV, representing a statistically significant decline (p < 0.0001). The mean ALSFRS-R score at baseline was 42.06, significantly declining to 36.72 at six months (Table 2). Similarly, the mean MUNE value declined from 16.36 at baseline to 13.37 at six months, demonstrating statistical significance (Table 3).

**Table 2:** Descriptive statistics of ALSFRS-R score at baseline and at six months *Paired t‑test (baseline vs six months). ALSFRS-R, Amyotrophic Lateral Sclerosis Functional Rating Scale–Revised; SD, standard deviation

ALSFRS-R score	Mean ± SD	Median (25th-75th percentile)	Range	P-value*
At baseline	42.06 ± 3.24	42 (40-45)	35-47	<0.0001
At six months	36.72 ± 4.89	37 (34-39.5)	25-45	

**Table 3:** Descriptive statistics of MUNE at baseline and at six months *Paired t‑test (baseline vs six months). MUNE, motor unit number estimation; SD, standard deviation

MUNE	Mean ± SD	Median (25th-75th percentile)	Range	P value*
At baseline	16.36 ± 5.22	16.4 (12.55-18.765)	5.4-28.4	<0.0001
At six months	13.37 ± 4.96	13.51 (10.78-15.45)	1.14-22.4	

**Figure 2:**
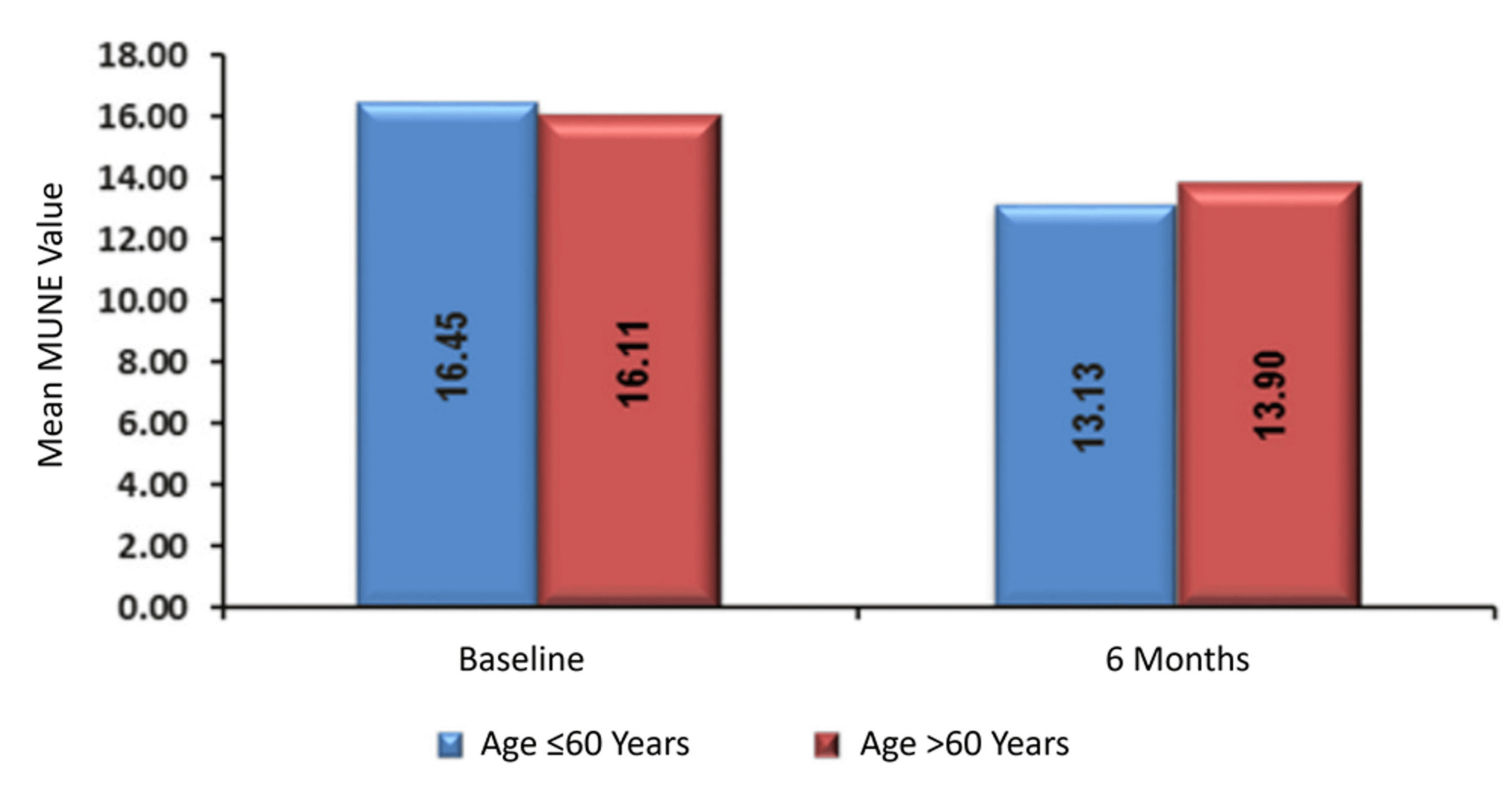
Association of MUNE with age of presentation. MUNE, motor unit number estimation

The mean baseline MUNE for patients aged ≤60 years was 16.45 ± 5.44, compared with 16.11 ± 4.88 for >60 years. This difference was not statistically significant (p = 0.862). At six months, the mean ± SD was 13.13 ± 5.46 for those aged ≤60 years and 13.9 ± 3.86 for those aged >60 years, again with no significant difference observed (p = 0.706; Table 4, Figure 2).

**Table 4:** : Association of MUNE with age of presentation *Independent samples t‑test (≤60 years vs >60 years). MUNE, motor unit number estimation; SD, standard deviation

MUNE		Participant age group		Total	P-value*
		≤60 years	>60 years		
At baseline	Mean ± SD	16.45 ± 5.44	16.11 ± 4.88	16.36 ± 5.22	0.862
	Median (25th-75th percentile)	16.66 (13.95-19.42)	15.75 (12.495-17.255)	16.4 (12.55-18.765)	
	Range	5.4-26	11.86-28.4	5.4-28.4	
At six months	Mean ± SD	13.13 ± 5.46	13.9 ± 3.86	13.37 ± 4.96	0.706
	Median (25th-75th percentile)	14.62 (9.458-15.55)	13.15 (11.55-14.85)	13.51 (10.78-15.45)	
	Range	1.14-20.8	9.41-22.4	1.14-22.4	

**Figure 3:**
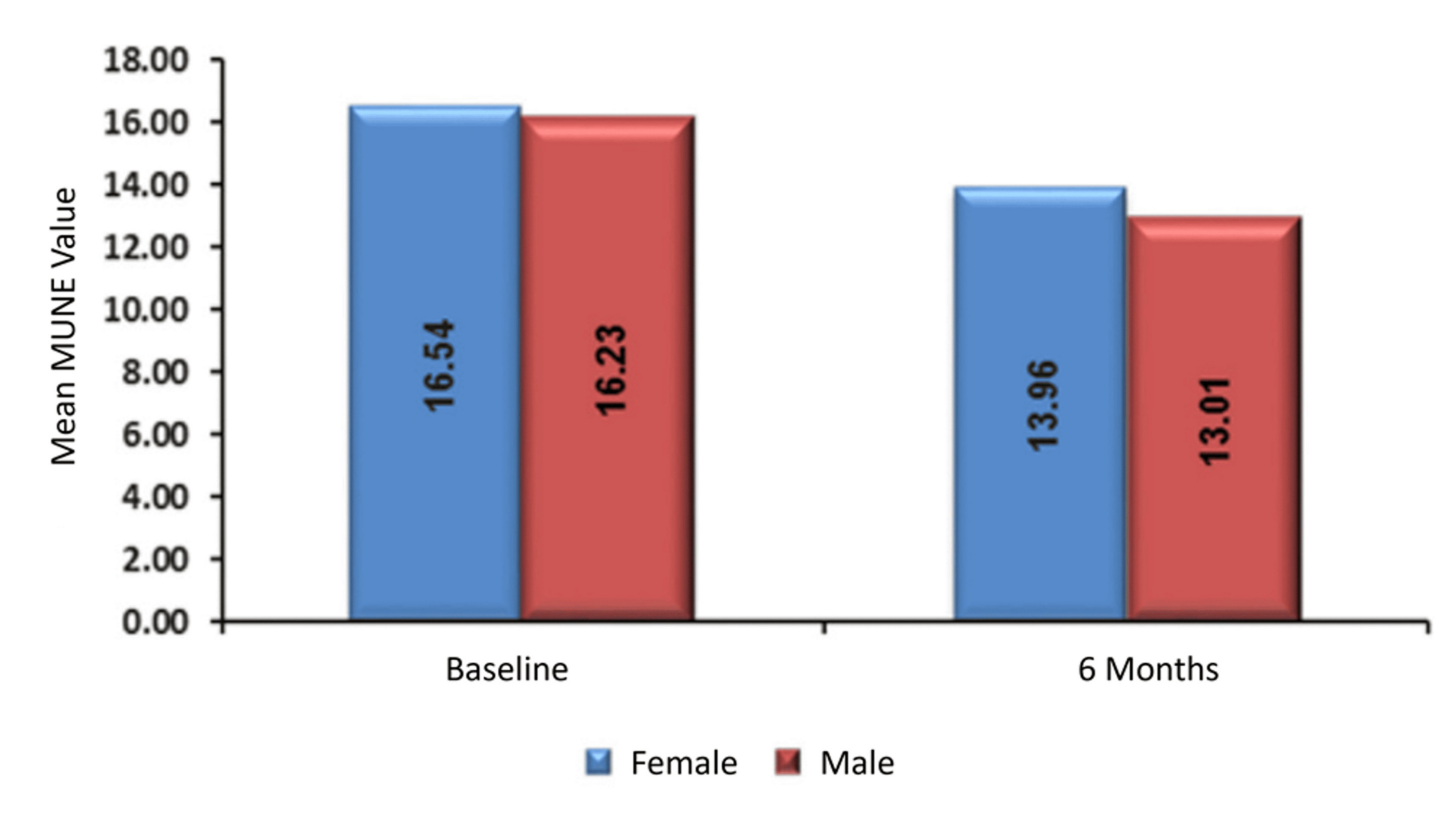
Association of MUNE with sex. MUNE, motor unit number estimation

The mean baseline MUNE value was 16.54 ± 4.23 for female participants and 16.23 ± 5.88 for male participants, with no significant difference between the two groups (p = 0.87). At six months, the mean MUNE was 13.96 ± 3.01 for females and 13.01 ± 5.9 for males, again with no statistically significant difference (p = 0.628; Table 5, Figure 3).

**Table 5:** Association of MUNE with sex *Independent samples t‑test (female vs male). MUNE, motor unit number estimation; SD, standard deviation

MUNE		Participant sex		Total	P-value*
		Female	Male		
At baseline	Mean ± SD	16.54 ± 4.23	16.23 ± 5.88	16.36 ± 5.22	0.87
	Median (25th-75th percentile)	16.98 (14.47-18.54)	16.13 (12.44-18.75)	16.4 (12.55-18.765)	
	Range	8.34-25.52	5.4-28.4	5.4-28.4	
At six months	Mean ± SD	13.96 ± 3.01	13.01 ± 5.9	13.37 ± 4.96	0.628
	Median (25th-75th percentile)	14.65 (12.735-15.15)	13.09 (9.752-16.4)	13.51 (10.78-15.45)	
	Range	8.8-19.72	1.14-22.4	1.14-22.4	

**Figure 4:**
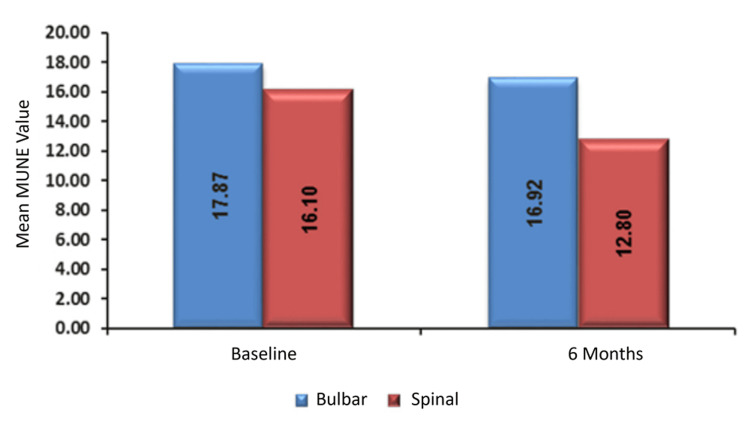
Association of MUNE with site of onset. MUNE, motor unit number estimation

No significant association was observed between MUNE values and site of disease onset either at baseline (p = 0.491) or at six months (p = 0.125). The mean baseline MUNE was 17.87 ± 7.48 for the bulbar-onset group and 16.1 ± 4.87 for the spinal-onset group. At six months, the bulbar group had a mean MUNE of 16.92 ± 4.39, while the spinal group had a mean of 12.8 ± 4.89, but these differences were not statistically significant (Table 6, Figure 4).

**Table 6:** Association of MUNE with site of onset *Independent samples t‑test (bulbar vs spinal onset). MUNE, motor unit number estimation; SD, standard deviation

MUNE		Site of onset		Total	P-value*
		Bulbar	Spinal		
At baseline	Mean ± SD	17.87 ± 7.48	16.1 ± 4.87	16.36 ± 5.22	0.491
	Median (25th-75th percentile)	16 (14.13-24.88)	16.45 (12.495-18.575)	16.4 (12.55-18.765)	
	Range	8.34-26	5.4-28.4	5.4-28.4	
At six months	Mean ± SD	16.92 ± 4.39	12.8 ± 4.89	13.37 ± 4.96	0.125
	Median (25th-75th percentile)	17.07 (13.32-20.665)	13.15 (9.5-15.29)	13.51 (10.78-15.45)	
	Range	12.75-20.8	1.14-22.4	1.14-22.4	

**Figure 5:**
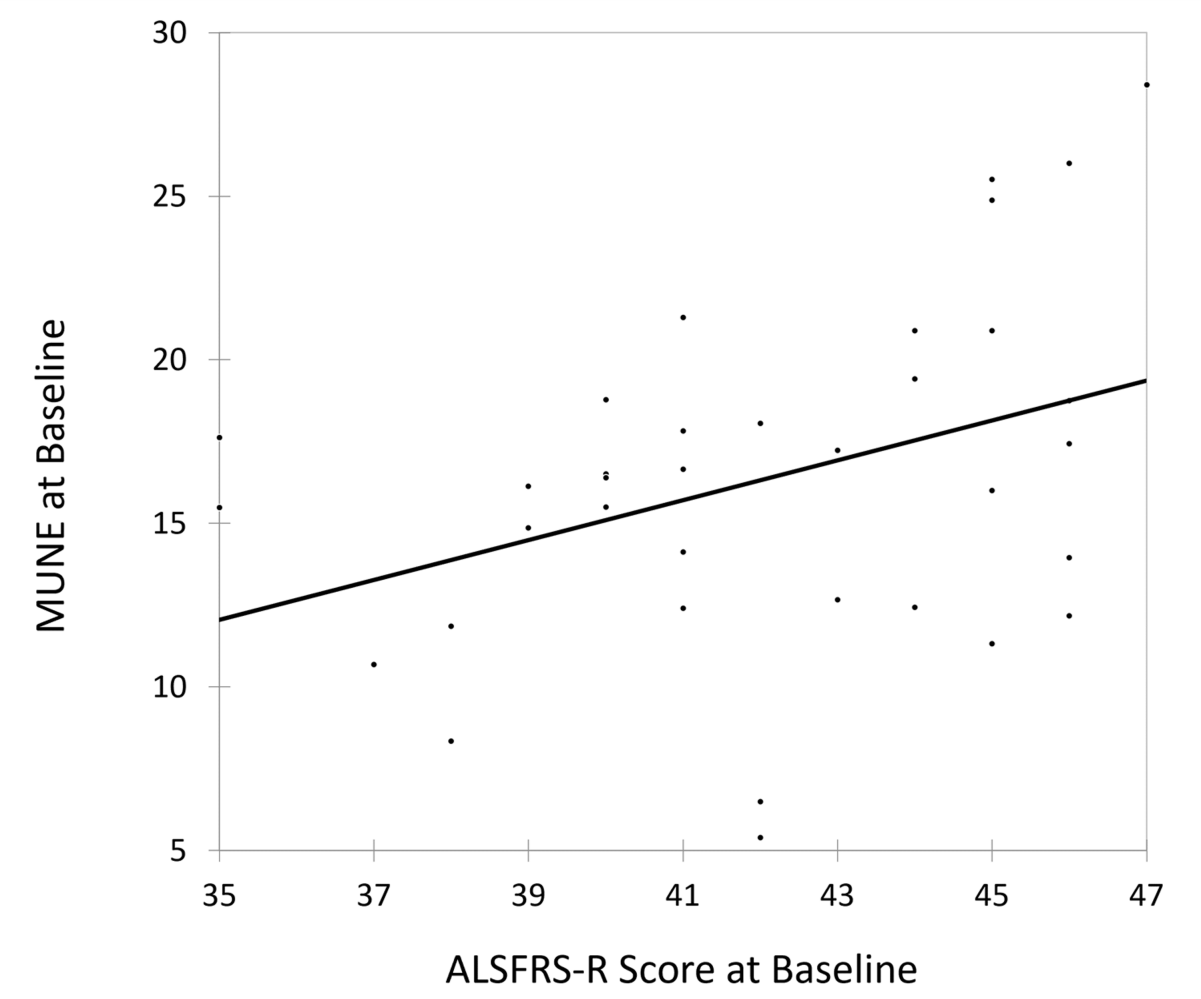
Correlation of ALSFRS-R score at baseline with MUNE at baseline. ALSFRS-R, amyotrophic lateral sclerosis Functional Rating Scale–Revised; MUNE, motor unit number estimation

**Figure 6:**
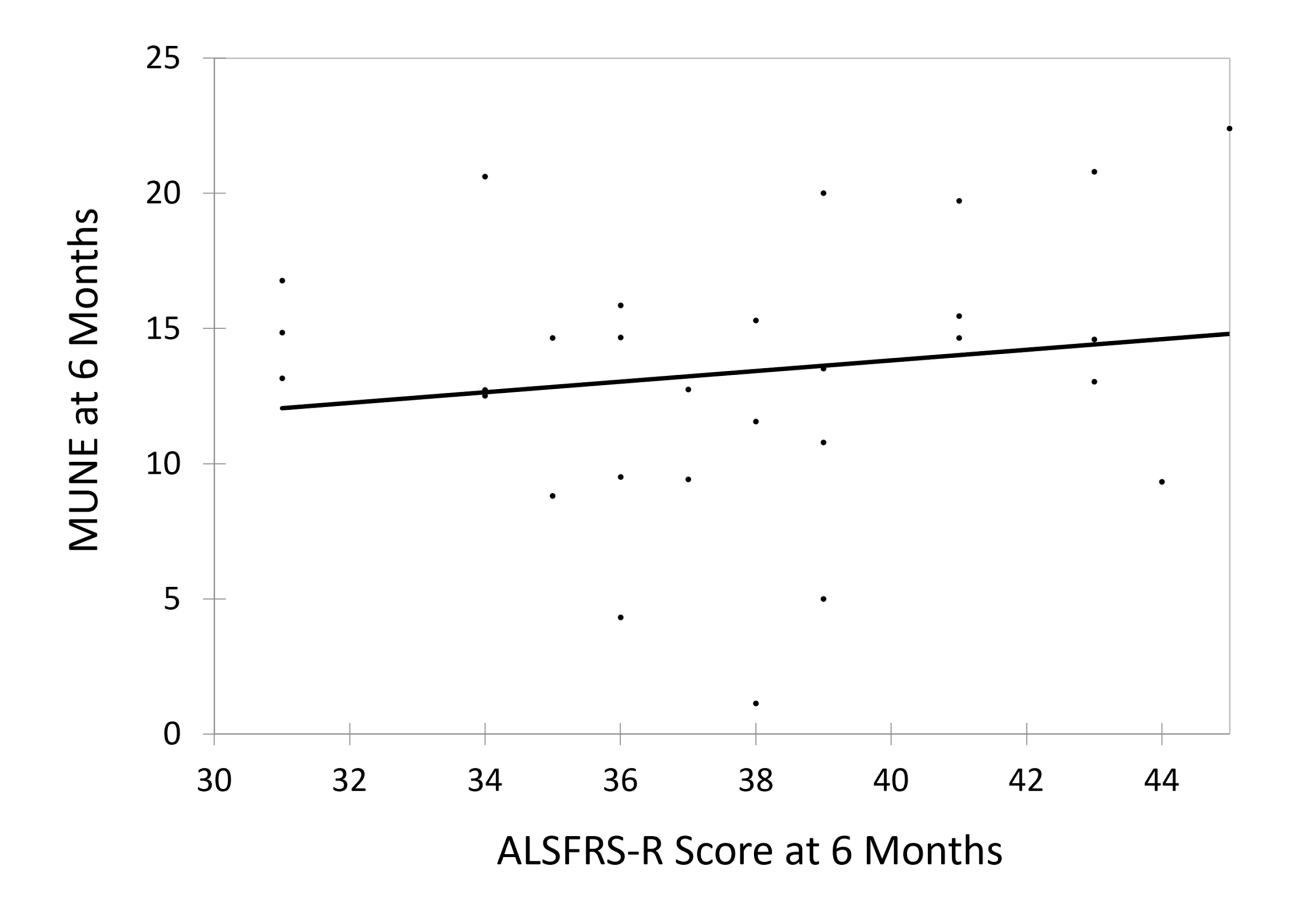
Correlation of ALSFRS-R score at six months with MUNE at six months. ALSFRS-R, amyotrophic lateral sclerosis Functional Rating Scale–Revised; MUNE, motor unit number estimation

Correlation analysis showed a weak positive association between the baseline ALSFRS-R score and baseline MUNE value, with a correlation coefficient of 0.379 (Figure 5). However, a very weak, non-significant positive correlation was noted at six months between ALSFRS-R and MUNE, with a correlation coefficient of 0.154 (Figure 6).

**Figure 7:**
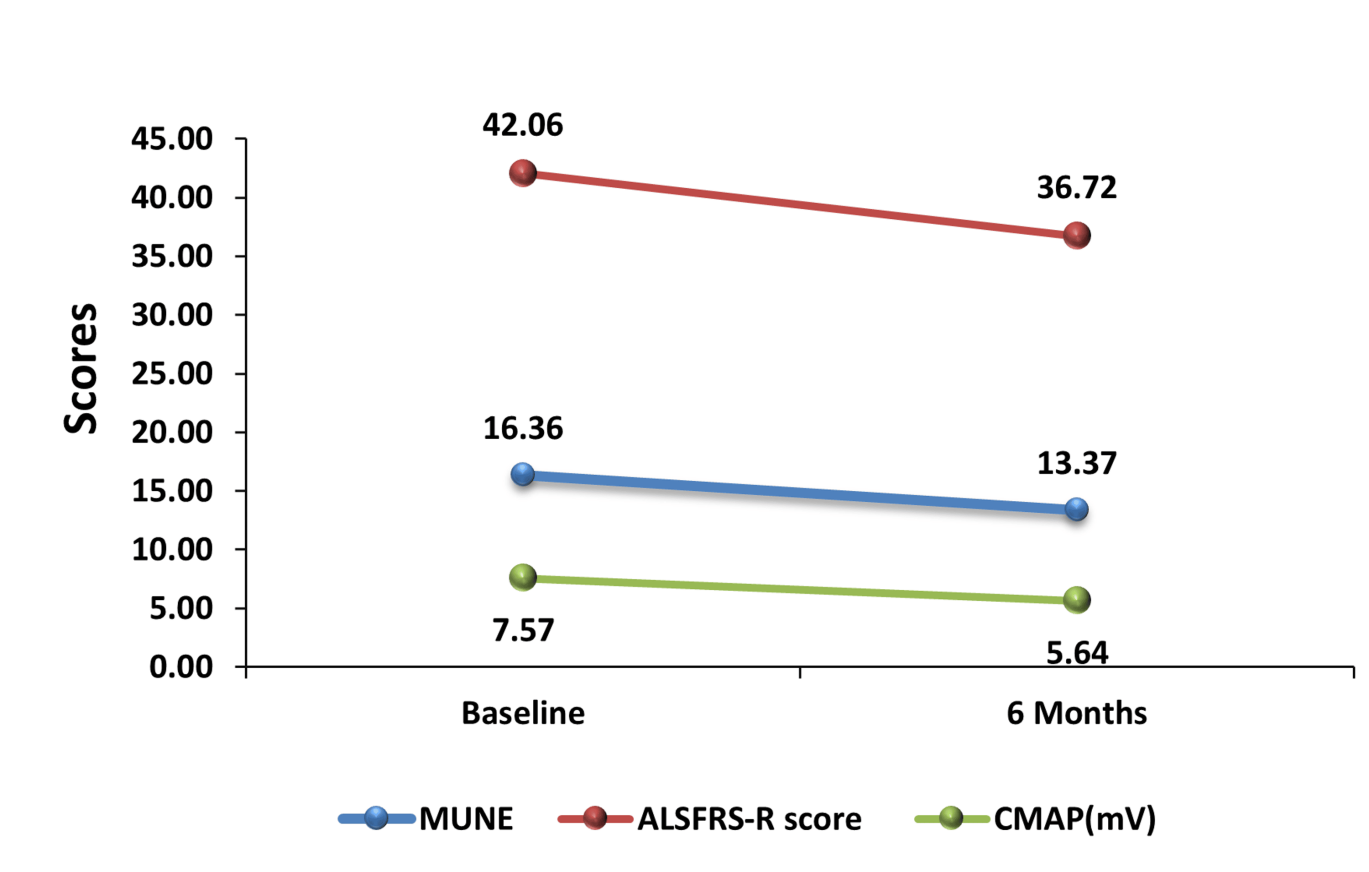
Trend of MUNE and ALSFRS-R score over time. ALSFRS-R, amyotrophic lateral sclerosis Functional Rating Scale–Revised; MUNE, motor unit number estimation

Time trend analysis comparing the rate of decline in ALSFRS-R and MUNE revealed that MUNE declined more rapidly and to a greater extent than ALSFRS-R over the six-month period (Figure 7). Table 7 shows a comparison of MUNE, ALSFRS-R scores, and CMAP values at baseline and at six months. At baseline, the mean MUNE was 16.36 ± 5.22, which decreased significantly to 13.37 ± 4.96 at six months (p < 0.0001). The ALSFRS-R score declined from a baseline mean of 42.06 ± 3.24 to 36.72 ± 4.89 at six months (p < 0.0001). Likewise, the mean CMAP value dropped from 7.57 ± 2.6 mV to 5.64 ± 2.1 mV, also showing a significant decline (p < 0.0001; Table 7).

**Table 7:** Descriptive statistics of MUNE and ALSFRS-R score *Paired t-test. ALSFRS-R, Amyotrophic Lateral Sclerosis Functional Rating Scale–Revised; CMAP, compound muscle action potential; MUNE, motor unit number estimation

Variables	Mean	Standard deviation	P-value
MUNE at baseline	16.36	5.22	<0.0001*
MUNE at six months	13.37	4.96	
ALSFRS-R score at baseline	42.06	3.24	<0.0001*
ALSFRS-R score at six months	36.72	4.89	
CMAP (mV) at baseline	7.57	2.6	<0.0001*
CMAP (mV) six months	5.64	2.1	

**Figure 8:**
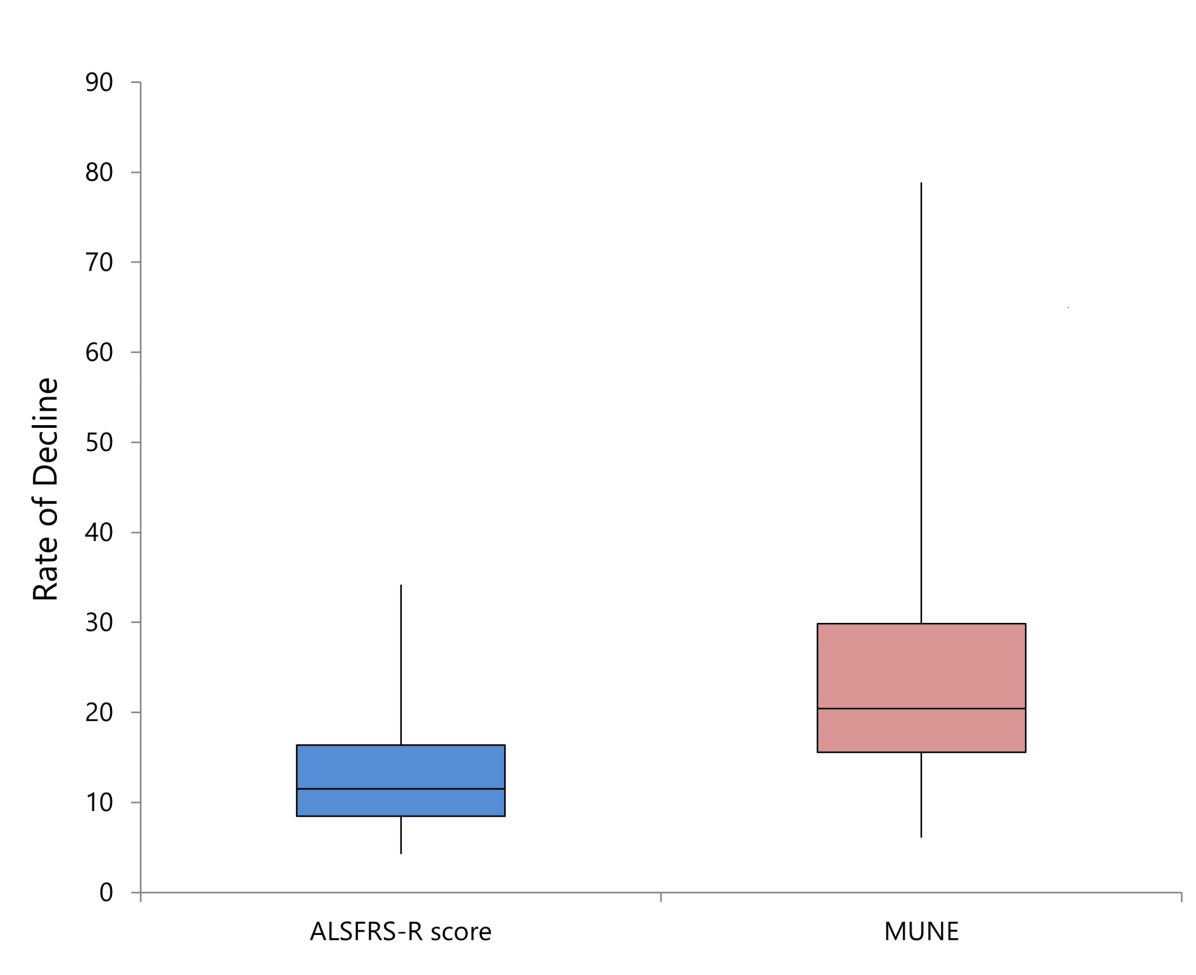
Comparison of rate of decline in ALSFRS-R score and MUNE. ALSFRS-R, amyotrophic lateral sclerosis Functional Rating Scale–Revised; MUNE, motor unit number estimation

The mean rate of decline for ALSFRS-R over the six-month period was 13.91, while the mean rate of decline for MUNE was 23.6. These findings indicate a greater rate of deterioration in MUNE compared to ALSFRS-R (Table 8, Figure 8).

**Table 8:** Comparison of rate of decline in ALSFRS-R score and MUNE *Paired t‑test (within‑patient difference in decline rates). ALSFRS-R, Amyotrophic Lateral Sclerosis Functional Rating Scale–Revised; MUNE, motor unit number estimation; SD, standard deviation

Assessment	Rate of decline			P-value*
	Mean ± SD	Median (25th-75th percentile)	Range	
ALSFRS-R score	13.91 ± 8.18	11.53 (8.496-16.364)	4.26-34.21	0.001
MUNE	23.6 ± 15.31	20.44 (15.563-29.871)	6.1-78.89	

## Discussion

ALS is the most common disorder affecting the anterior horn cells [1]. Despite extensive research, the heterogeneous nature of ALS progression has prevented the development of a reliable, practical, and validated prognostic model [11]. The need for an effective biomarker to assess disease progression in both patients and clinical trials has led to exploring methods to quantify motor unit loss. In this context, the MUNE technique was introduced in the 1970s as a potential surrogate marker of motor neuron degeneration [12]. This study aimed to evaluate whether MUNE could serve as a biomarker of disease progression in patients with ALS. In parallel, we compared MUNE with the ALSFRS-R to assess whether MUNE offers advantages in sensitivity over traditional clinical measures.

Our study’s mean ALSFRS-R score declined significantly from 42.06 ± 3.24 at baseline to 36.72 ± 4.89 at six months (p < 0.0001), reflecting disease progression. This negative correlation is consistent with findings from earlier studies that demonstrated worsening ALSFRS-R scores over time in patients with ALS [3,4]. Chiò et al. also reported that patients with lower ALSFRS-R scores had poorer prognoses than those with higher scores [13].

We additionally assessed ALSFRS-R scores by site of symptom onset. At baseline, the mean ALSFRS-R score was 43 ± 3.39 for patients with bulbar-onset ALS and 41.9 ± 3.25 for those with spinal-onset disease. At six months, scores declined to 35.6 ± 6.77 and 36.93 ± 4.6, respectively. However, there was no statistically significant association between ALSFRS-R scores and the site of onset at either time point (p = 0.491 at baseline, p = 0.586 at six months). This may reflect limitations of the ALSFRS-R, such as inconsistent scoring in the respiratory domain [14] and a lack of standardized administration procedures, which may limit its ability to capture functional decline accurately [15].

The mean MUNE value decreased significantly from 16.36 ± 5.22 at baseline to 13.37 ± 4.96 at six months (p < 0.0001), supporting its potential utility as a progression marker. Given that MUNE has been shown to decrease with age [16], we conducted subgroup analyses to examine whether age influenced MUNE values in our population. At baseline, the mean MUNE was 16.45 ± 5.44 in patients aged ≤60 years and 16.11 ± 4.88 in those aged >60 years, with no significant difference (p = 0.862). At six months, the values were 13.13 ± 5.46 and 13.9 ± 3.86, respectively, also without statistical significance (p = 0.706). These results suggest that age did not significantly affect MUNE in this ALS-only cohort, likely due to the overriding effect of disease-related motor neuron loss.

We also performed subgroup analyses by sex. At baseline, the mean MUNE was 16.54 ± 4.23 in female participants and 16.23 ± 5.88 in male participants (p = 0.87). At six months, these values declined to 13.96 ± 3.01 and 13.01 ± 5.9, respectively, again without significant difference (p = 0.628). Similarly, site of symptom onset did not significantly impact MUNE values. At baseline, the bulbar group had a mean MUNE of 17.87 ± 7.48, while the spinal group had a mean of 16.1 ± 4.87. At six months, MUNE values were 16.92 ± 4.39 and 12.8 ± 4.89, respectively (p = 0.491 at baseline, p = 0.125 at six months). These findings highlight that MUNE was reduced across all subgroups, including those without prominent limb weakness, indicating that motor unit loss likely precedes overt clinical weakness.

A significant reduction in MUNE over six months was also observed in studies by Jagtap et al. and Boekestein et al., both of which demonstrated greater sensitivity of MUNE compared to ALSFRS-R or CMAP for detecting early motor neuron loss [17,18]. The correlation between MUNE and ALSFRS-R scores in our study was weak and not statistically significant at either baseline or six months. However, both measures declined over time, and the rate of decline in MUNE was notably greater.

The mean rate of decline in ALSFRS-R score was 13.91 ± 8.18, while the mean decline in MUNE was 23.6 ± 15.31. This difference was statistically significant (p = 0.001), indicating that MUNE may be a more sensitive progression marker than ALSFRS-R. Similar trends have been reported by Xiao-Xuan Liu et al. and Jagtap et al., who also found MUNE to decline significantly faster than ALSFRS-R over a six-month period [17,19].

Limitations

This study has several limitations. First, it lacked a healthy control group, making it difficult to determine whether the decline in MUNE is specific to ALS or also occurs with aging or other comorbidities. Second, the relatively small sample size may limit the generalizability of the findings. Third, because the study was conducted at a tertiary care center, referral bias may have influenced the clinical characteristics of the enrolled population.

## Conclusions

Our study demonstrated a significant reduction in MUNE and ALSFRS-R scores over six months, indicating disease progression. However, the rate of decline in MUNE was significantly greater than that observed in ALSFRS-R scores, suggesting that MUNE may detect early motor neuron loss more sensitively than traditional clinical scales. Using MUNE in clinical practice and research could enhance the precision of prognostication, improve patient stratification in clinical trials, and potentially enable earlier interventions by capturing subclinical motor unit loss before overt functional decline occurs. Further large-scale and longitudinal studies are warranted to validate these findings and establish standardized protocols for implementing MUNE in routine ALS care and therapeutic trials.
